# Effective Removal of Crystal Violet Dye Using Neoteric Magnetic Nanostructures Based on Functionalized Poly(Benzofuran-*co*-Arylacetic Acid): Investigation of the Adsorption Behaviour and Reusability

**DOI:** 10.3390/nano11030679

**Published:** 2021-03-09

**Authors:** Iolanda-Veronica Ganea, Alexandrina Nan, Călin Baciu, Rodica Turcu

**Affiliations:** 1 Physics of Nanostructured Systems Department, National Institute for Research and Development of Isotopic and Molecular Technologies, 400293 Cluj-Napoca, Romania; iolanda.ganea@itim-cj.ro; 2 Faculty of Environmental Science and Engineering, “Babeș-Bolyai” University, 400294 Cluj-Napoca, Romania; calin.baciu@ubbcluj.ro

**Keywords:** magnetic nanoparticles, crystal violet, folic acid, poly(benzofuran-*co*-arylacetic acid), adsorption kinetic studies

## Abstract

Synthetic dyes represent a significant class of contaminants released in the environment. Crystal violet is a triarylmethane dye used in several fields such as printing inks, the textile or paper industries, as well as in cell histology. Coating magnetic nanoparticles with functionalized polymers has been proved to improve their efficiency, offering unique properties for applications in wastewater treatment. The current paper focuses on preparing and characterising magnetic core-shell nanoparticles coated with poly(benzofuran-*co*-arylacetic acid) functionalized with folic acid as an organic shell. The new polymer-based magnetic nanostructures were applied for crystal violet extraction from aqueous solutions. The nanostructures were structurally and morphologically investigated by Fourier-transform infrared (FTIR) spectroscopy and transmission electron microscopy (TEM). While thermal and magnetic properties of the magnetic nanostructures were determined by thermogravimetric analysis (TGA) and magnetization measurements (VSM). At the same time, crystal violet concentrations were determined by UV-VIS spectroscopy. The influence of initial dye concentration and contact time on the removal efficiency has been studied to achieve the optimum adsorption conditions. The dye adsorbent neoteric magnetic nanostructure was easily desorbed and reused, the adsorption capacity decreasing from 100% to 97.63% in the first five cycles, reaching a minimum of 88.74% after the 10th recycling step.

## 1. Introduction

Nowadays, dyes are widely used in many applications like plastics and paper, textiles, cosmetics, pharmaceuticals, food, dyestuffs, etc. [1]. Annually, more than 150,000 tons of residual dyes are released as industrial wastes worldwide, without any pre-treatment [2]. Around 1500 million L of dye-contaminated effluents are discharged daily in different environmental factors, posing a significant threat to both biota and human health [3,4]. Thus, synthetic dyes have become a serious concern for both toxicologists and environmental specialists. Several physical, biological, and chemical treatment techniques such as flocculation, filtration, electrodialysis, oxidation, biosorption have been used to treat these effluents [3,5,6]. However, each of them has its drawbacks (long contact time, toxic secondary products generation, price etc.) and in certain situations, they are not very effective due to the complex chemical composition of the dye-contaminated waters.

Crystal violet (CV) is a synthetic cationic dye variously called aniline violet, pyoctanin, or Gentian violet. It is often found in industrial effluents due to its massive applications in medicine as an anthelmintic, antimicrobial agent, biological stain, bacteriostatic, and colourant in the textile, detergents or fertilizers industries and printing facilities [7]. Nevertheless, crystal violet is considered a biohazard substance because it is toxic, clastogenic, persistent, potentially carcinogen, it promotes tumour growth and acts as a mitotic poison. The acute and chronic health effects due to ingestion or inhalation include skin or eyes irritation, blepharospasm, gastrointestinal and respiratory tract irritation, nausea, abdominal pain, paralysis, peritonitis, weight loss, ataxia, pulmonary edema, adverse reproductive effects or birth defects and cancer. High concentrations of crystal violet in effluents can reduce or stop water bodies’ oxygenation capacity and affect the photosynthesis process and biological activity in aquatic biota [8,9]. Thus, crystal violet removal from surface waters or wastewaters is mandatory. The adsorption process represents one of the most promising methods for crystal violet removal. In the past decades, the following adsorbents were used to extract crystal violet from wastewaters: activated carbon [10], ash [11], graphenes [12], biomass [13], agricultural wastes [14], acid-based hydrogels [15], magnetite-coated biochar [16], clays [8], silica [17], zeolites [7], manganese oxide-based materials [18], nanorods [19], nanomagnetic iron oxide [20], magnetic calcium ferrite nanoparticles [21], etc. Recently, the interest in finding adequate “environmentally-friendly” adsorbents and improving their adsorption capacities has increased exponentially. Magnetic separation employing magnetic nanoparticles (MNPs) sorbents gained interest due to their ease in retrieving the adsorbent from the aqueous phase [22]. Folic acid (FA) is an antimutagenic, nontoxic pteridine derivative very abundant in plants (citrus fruits, leafy green vegetables, beans etc.) or liver [23] and has various functional groups (including carbonyl, carboxyl, hydroxyl and amines), able to extract many contaminants from aqueous solutions [24].

In the present paper, polymer-functionalized MNPs with core-shell structure based on poly(benzofuran-*co*-arylacetic acid) (**PBAAA**) functionalized with FA (**MNP@PAAA-FA**) were synthesized and applied as a novel adsorbent material for CV elimination from aqueous solutions. The functionalized polymer (**PAAA-FA**) required for covering the MNP surface was obtained through opening the lactone ring, present in the polymer structure, by the amino group of FA. In this article, folic acid immobilized on the **PBAAA** polymer as a dye absorbent was investigated for the first time. The new magnetic nanostructures (**MNP@PAAA-FA**) were structurally, morphologically, and magnetically investigated, while CV concentrations were measured by UV-VIS spectroscopy. The magnetic nanostructures present good dispersibility in water and easy separation after adsorption. The influence of two adsorption parameters (preliminary dye concentration and interaction time) has been analysed to establish the most advantageous sorption conditions. Considering the magnetic properties of the nanostructures, their recyclability was also important to be tested.

## 2. Materials and Methods

### 2.1. Materials

The syntheses of the polymer **PBAAA** and magnetite nanoparticles **MNP** were achieved based on previously described methods [25,26,27,28,29]. All the other reagents used in this study were purchased from Sigma Aldrich (St. Louis, MO, USA) and Alfa Aesar by ThermoFisher Scientific (Kandel, Germany) and did not require further purification.

### 2.2. Preparation of Core-Shell Magnetic Nanostructures Based on Poly(Benzofuran-co-Arylacetic Acid) Functionalized with Folic Acid (**MNP@PAAA-FA**)

A solution of **PBAAA** (1 g) and **FA** (0.50 g) was prepared in a mixture of dimethyl sulfoxide (DMSO) (80 mL) and methanol (40 mL) and refluxed for 48 h. Afterwards, the solvent was evaporated under vacuum to obtain 1.34 g of functionalized polymer **PAAA-FA**. Furthermore, **PAAA-FA** (0.90 g) was added to a very well-dispersed suspension of **MNP** (0.75 g) in 15 mL of deionized water and magnetically stirred for 24 h at room temperature. The resulting nanostructure, **MNP@PAAA-FA**, was separated magnetically and rinsed with water and methanol, ensuing 1.2 g of functionalized magnetic nanoparticles.

### 2.3. Instrumentation

Infrared absorption spectra (400–4000 cm^−1^) were analyzed on a pressed tablet prepared from magnetic powder enclosed in KBr, with a JASCO FTIR-6100 spectrophotometer (JASCO Deutschland GmbH, Pfungstadt, Germany). Hitachi H9000NAR (Hitachi Ltd., Tokyo, Japan) and 1010 JEOL transmission electron microscopes (JEOL Ltd., Tokyo, Japan) were used to establish the MNPs’ morphology. A cryogenic vibrating sample magnetometer (Cryogenic Ltd., London, UK) was used to perform magnetic measurements at room temperature. Thermogravimetry measurements were achieved using TA Instruments SDT Q 600 equipment (TA Instruments Inc., New Castle, DE, USA), in air, at a heating rate of 10 °C min^−1^ within a range from 30 °C to 800 °C. X-ray Powder Diffraction (XRPD) measurements were performed with a Bruker D8 Advance X-ray diffractometer (Bruker, Billerica, MA, USA), with a Ge (111) monochromator for Cu-Kα1 radiation (λ = 1.5406 Å) having the source power of 40 kV and 40 mA, at room temperature and with a LynxEye position sensitive detector (Bruker, Karlsruhe, Germany). The magnetite nanostructures were placed into a sample holder and the measurement was continuously run. The experiment was recorded by monitoring the diffraction pattern appearing in the 2θ range from 10 to 90 with a scan speed of 1° min^−1^ and a scan step of 0.02°. N_2_ adsorption–desorption isotherms (registered at −196 °C) were used to calculate the total surface area (S_t_), pore volume (V_p_) and pore radius (R_m_) using the BET method for S_t_ and Dollimore—Heal model for V_p_ and R_m_. The isotherms were recorded using a Sorptomatic 1990 apparatus (Thermo Electron Corporation, Waltham, MA, USA). Samples were degassed at 70 °C for 5 h at a pressure of 1 Pa prior determination, to remove the physisorbed impurities from the surface. UV-VIS spectra were recorded in the 190–900 nm wavelengths range at a spectral resolution of 1 nm with a Jasco V-550 UV-VIS Spectrophotometer (JASCO Deutschland GmbH), equipped with a double-beam photometer and a single monochromator, using 10 mm length quartz cells. Sample aliquots were collected before and after performing adsorption tests and CV, absorbance was measured at 591 nm wavelength.

### 2.4. Batch Adsorption Experiments

The batch sorption studies involved the preparation of 38 different CV stock solutions (ranging from 0.45 to 500 mg L^−1^) using ultrapure water produced with a Milli-Q (Millipore, Bedford, MA, USA). Adsorption experiments were completed at room temperature in an HLC Heating-ThermoMixer MHR11 (DITABIS Digital Biomedical Imaging Systems AG, Pforzheim, Germany) at a shaking frequency of 700 rpm. **MNP@PAAA-FA** was magnetically withdrawn from the aqueous solutions, and the remaining amount of CV was measured by UV-VIS analysis. We analyzed two different adsorption conditions, such as preliminary CV concentration and interaction time, to study the CV removal efficiency. The removal efficiencies and sorption capacities (Equations (1) and (2)) of the newly synthesized nanostructures **MNP@PAAA-FA** were calculated based on the next equations:

(1) $$R\;\left(\%\right)=\frac{C_{i}-C_{f}}{C_{i}}\times 100$$

(2) $${q\;(\mathrm{mg}\;g}^{-1})=\frac{\left(C_{i}-C_{f}\right)\times V}{w}$$

where: R is the adsorption efficiency (%), C_i_ and C_f_ are the initial and final CV concentrations (mg L^−1^), q is the sorption capacity (mg g^−1^), V is the volume of solution (L), and w is the amount of adsorbent (g).

#### 2.4.1. Equilibrium Study on CV Dye Adsorption by **MNP@PAAA-FA**

The equilibrium sorption study was achieved using 3 mL of CV solutions of 38 different concentrations (0.45–500 mg L^−1^) for an adsorbent amount of 3.33 g L^−1^ and 30 min contact time. Experimental data were fitted on both the linear and non-linear forms of Langmuir, Freundlich, Temkin, Dubinin-Radushkevich and Khan isotherm models (Table S1) [30,31,32,33,34]. Equation (3) defines one of the most important attributes of the Langmuir isotherm, namely the separation factor (R_L_), which shows the type of adsorption process and the isotherm profile:

(3) $$R_{L}=\frac{1}{{1+K}_{L}{\times C}_{i}}$$

where: R_L_ is the separation factor, and K_L_ is the Langmuir constant (L mg^−1^).

According to [35,36], the adsorption mechanism can be categorized as: irreversible (if R_L_ = 0), favourable (if 0 < R_L_ < 1), linear (if R_L_ = 1) and unfavourable (if R_L_ > 1).

Descriptive statistics was performed using Origin v.2018 (OriginLab Corporation, Northampton, MA, USA) and Anscombe’s quartet model was developed by using Anaconda v.2–2.4.0 (*numpy* library) (Anaconda, Inc., Austin, TX, USA) and Python programming (Python v. 3.9.2, The Python Software Foundation, Beaverton, OR, USA).

#### 2.4.2. Kinetic Studies on CV Dye Adsorption by **MNP@PAAA-FA**

The kinetics sorption study was conducted using 100 mg of **MNP@PAAA-FA** and 30 mL of 6 initial CV solutions: 0.50 mg L^−1^, 1 mg L^−1^, 10 mg L^−1^, 100 mg L^−1^, 250 mg L^−1^ and 500 mg L^−1^. The mixtures were shaken for 3 h at room temperature, with a speed of 700 rpm. Samples were withdrawn from each solution after 5, 15, 30, 45, 60, 90, 120, 150 and 180 min. Three kinetic models were applied to define CV adsorption behaviour on **MNP@PAAA-FA**: pseudo-first-order, pseudo-second-order and Weber-Morris intra-particle diffusion models (Table S2) [37,38,39].

#### 2.4.3. Regeneration Study of **MNP@PAAA-FA**

Desorption experiments were performed to check the reusability of **MNP@PAAA-FA**. Ten adsorption-desorption cycles were conducted in this regard. 16.6 mg magnetic nanostructures were introduced in 5 mL of 2 mg L^−1^ CV solution and shaken for 45 min at 700 rpm. Afterwards, the synthesized nanostructures were separated from the solution with an external magnetic and subsequently washed with 20 mL of 10% methanol solution of acetic acid and 5 mL absolute ethanol. The remaining CV solutions were investigated via UV-VIS analysis.

## 3. Results

### 3.1. Synthesis and Characterization of Magnetic Nanostructure Based on **MNP@PAAA-FA**

The polymer synthesized from 4-hydroxymandelic acid as a sustainable resource is prepared through a remarkably green synthesis by merely heating the monomer in a non-catalytic and solvent-free technique. The synthesis occurs by a poly-Friedel Crafts alkylation, leading to a polycarboxylic acid, **PBAAA,** which contains two other reactive groups—phenol and lactone units in its structure. The pendant lactone rings from **PBAAA** chain are exploited to link **FA** by the free amino group. The resulting highly functionalized polymer **PAAA-FA** is suitable for coating the magnetite nanoparticles surface.

**Scheme 1:**
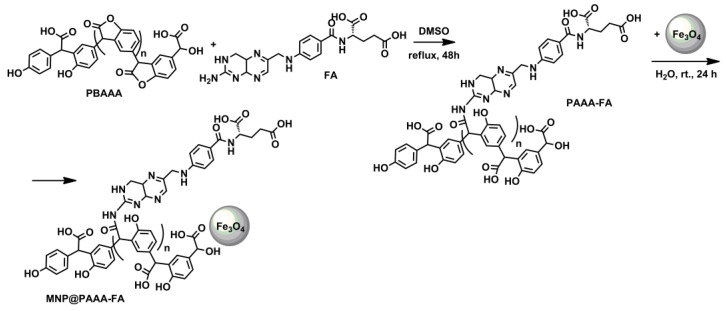
Preparation of **MNP@PAAA-FA**.

The preparation of core-shell nanostructures based on **PBAAA** functionalized with **FA** consists of four steps: (i) **PBAAA** synthesis by Friedel Crafts alkylation of 4-hydoxymadelic acid; (ii) binding of **FA** on the polymer chain, through opening the lactone ring by the free amino group present in **FA** structure; (iii) **MNP** preparation through co-precipitation method; (iv) adsorption of the modified polymer on the surface of the nanoparticles resulting in the new magnetic nanostructure—**MNP@PAAA-FA**. The first and third steps are defined in the literature. The second step, namely the lactone ring-opening by the amino group, is a chemical reaction that usually not involves the use of a catalyst. Still, due to the **FA** insolubility in water, this reaction was carried out in dimethyl sulfoxide. The last step in preparing **MNP@PAAA-FA** nanostructures is the simple adsorption of the modified polymer onto the surface of **MNP** (Scheme 1). The crystalline structure of the magnetite nanoparticles is characterized by XRD, as shown in Figure S1. The diffraction peaks of (220), (311), (400), (422), (511) and (440) reflect the magnetite crystal with a cubic spinel structure [40].

#### 3.1.1. Infrared Spectroscopy

**Figure 1:**
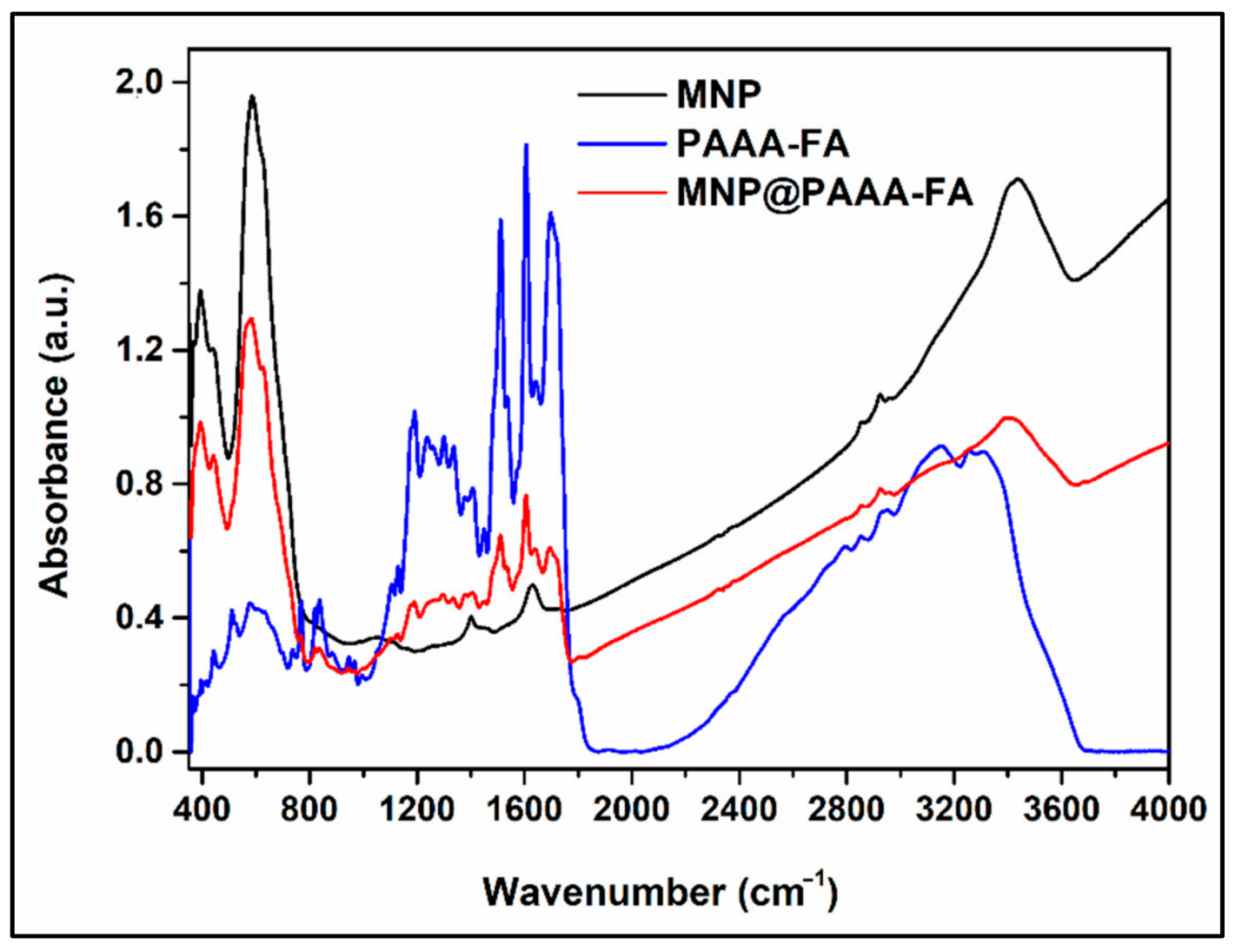
FTIR spectra of uncoated **MNP**, **PAAA-FA** and the **MNP@PAAA-FA** magnetic nanostructure collected between 400 and 4000 cm^−1^ using 1.92 mg of sample as pressed pellets embedded in KBr.

Figure 1 shows considerable changes in the **MNP** FTIR spectrum after the attachment of **PAAA-FA**. The intense absorption band specific to Fe-O bond emerges in the FTIR spectrum of **MNP** at the value of 581 cm^−1^, but the same absorption band occurs split in the FTIR spectrum of **MNP@PAAA-FA** at 585 cm^−1^ and 630 cm^−1^ wavenumbers. A principal effect of nanosized iron oxide particles when the polymer got adsorb on the surface is the unbinding of many bonds from the surface atoms, resulting in the rearrangement of localized electrons on the surface of the magnetite nanoparticles. The absorption band situated at 1704 cm^−1^ in the FTIR spectra of **MNP@PAAA-FA** is assigned to the C=O bond from the carboxyl group. The band located at 1646 cm^−1^ is attributed to the C=O bond (Amide I), and the strong absorption band at 1608 cm^−1^ is ascribed to the N-H bending and N-C=O group (Amide II), both bands appearing as a consequence of the covalent linkage of **FA** on the polymer chain. The aromatic hydrocarbons abundant in the structure of the functionalized polymer, show absorptions bands in the regions of 1400–1500 cm^−1^ and 1585–1610 cm^−1^ due to the C-C stretching vibrations present in the aromatic ring. The absorption band correlated with the C-O stretching bond is found at 1297 cm^−1^. The medium absorption band at 1187 cm^−1^ is attributed to the C-N bond. The emergence of the large absorption band between 3147 cm^−1^–3300 cm^−1^ can be ascribed to the O-H, N-H and C-H stretching vibrations from the aromatic ring. The presence of both the absorption bands specific to the magnetite and those specific to the functionalized polymer in the FTIR spectrum of the **MNP@PAAA-FA** indicates the formation of the core-shell magnetic nanostructure.

#### 3.1.2. Transmission Electron Microscopy

**Figure 2:**
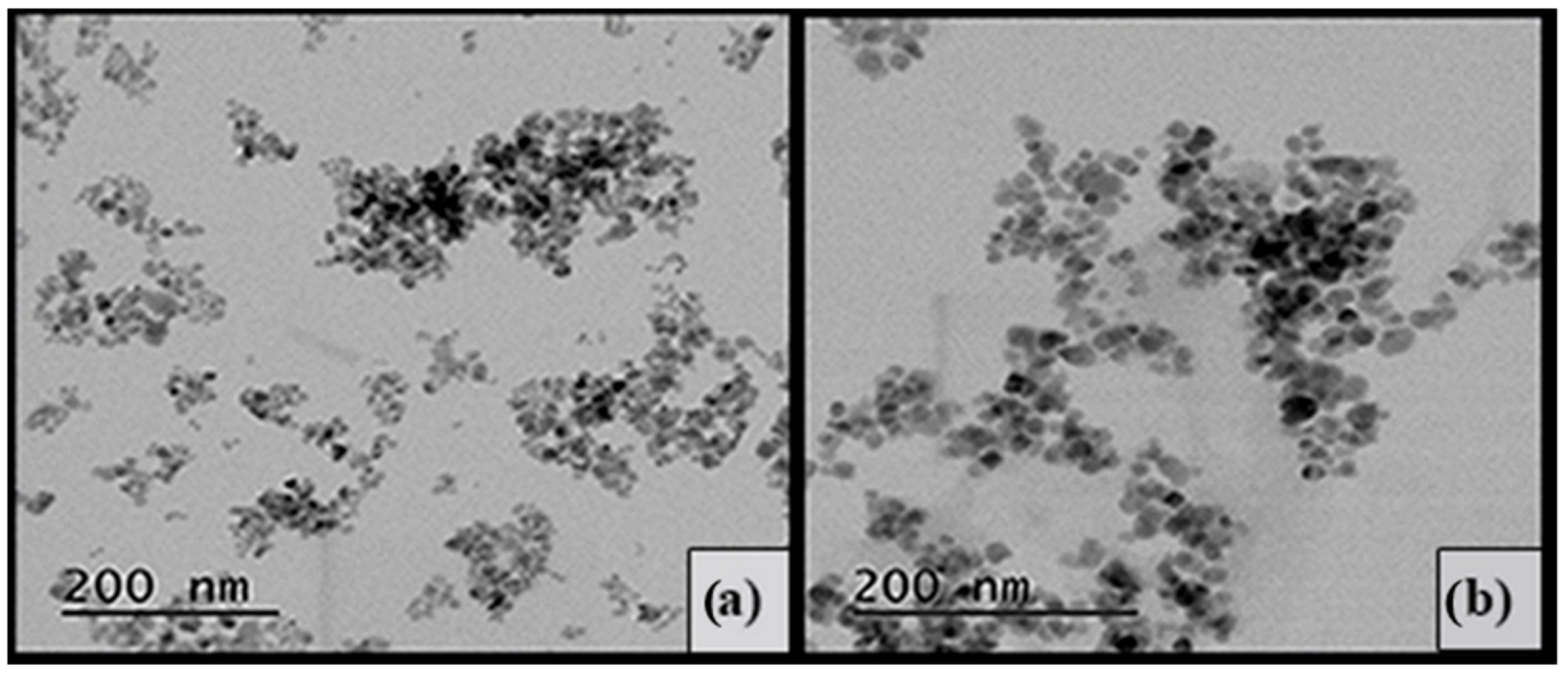
TEM image of uncovered **MNP** (**a**) and magnetic nanostructure **MNP@PAAA-FA** (**b**) measured using a 10 µL deposited drop of a suspension of each sample that was dried on a copper grid coated with a thin carbon film.

TEM investigations of **MNP** and **MNP@PAAA-FA** (Figure 2) revealed that both of them have spherical morphology. The uncovered **MNP** (Figure 2a) have a mean particles size of about 12–15 nm, while in the case of **MNP@PAAA-FA** (Figure 2b), the particles size increased to 20–27 nm, due to the coating process. It can also be observed that the newly synthesized magnetic nanostructures (**MNP@PAAA-FA**) are relatively well dispersed. Even if in the TEM images of **MNP@PAAA-FA** they look relatively well distributed, during their use as dye absorbent, a slight aggregation can be noticed.

#### 3.1.3. TGA Measurements

**Figure 3:**
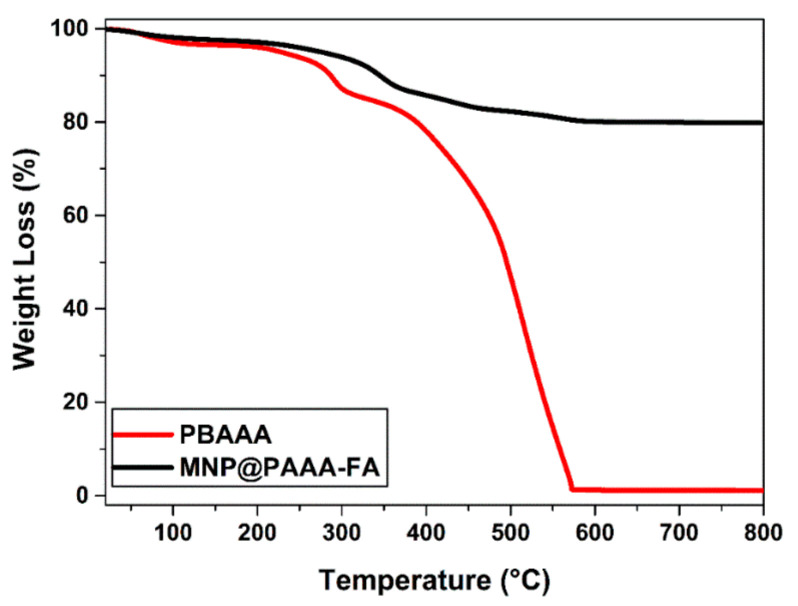
TGA curve of the starting polymer **PBAAA** and magnetic nanostructure **MNP@PAAA-FA** each performed in air at a heating rate of 10 °C min^−1^ between 30 °C and 800 °C.

Figure 3 reveals the TGA curves of **PBAAA** and the newly magnetic nanostructures **MNP@PAAA-FA**, starting from room temperature up to 800 °C in air. **PBAAA** records an initial mass loss of 8% at around 270 °C associated with the removal of intra- and inter-layer water molecules and the decarboxylation process, followed by an 82% reduction at 340 °C and in the end a 100% decomposition at 580 °C. On the other hand, **MNP@PAAA-FA** suffers a total mass loss of 21%, characterized by a small initial slope at around 300 °C, most probably corresponding to the organic shell degradation of the **MNP**, after which equilibrium is reached at 500 °C. The 20% decrease in mass for the magnetic nanostructure **MNP@PAAA-FA** is due to the loss of organic part, **PAAA-FA** proving the successful **MNP** coating with **PAAA-FA**.

#### 3.1.4. Magnetic Properties

**Figure 4:**
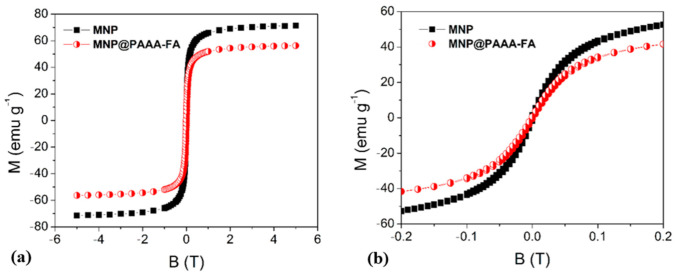
Magnetization vs. applied magnetic field for uncoated **MNP** and magnetic nanostructures **MNP@PAAA-FA** (**a**) and magnetization cycles at the low magnetic field (**b**) measured at room temperature using 0.02 g of dried sample.

The typical saturation magnetization behaviour of **MNP** and the newly synthesized magnetic nanostructures **MNP@PAAA-FA** measured at room temperature is described in Figure 4. The curves shape indicates a superparamagnetic behavior for **MNP** (68.60 emu g^−1^) and also highlights a decrease of the saturation magnetization for **MNP@PAAA-FA** (58.70 emu g^−1^) due to the loading of **MNP** with the **PAAA-FA** coating, which provides a higher non-magnetic portion in the resulting nanomaterial.

### 3.2. Batch Adsorption Experiments

#### 3.2.1. **MNP@PAAA-FA** Porosity Analysis

The N_2_ adsorption–desorption isotherms are of type IV (Figure S2) with hysteresis on the desorption branch indicating mesopores’ presence in both samples [41]. The surface area is 84 m^2^ g^−1^ for **MNP** and decreases to 78 m^2^ g^−1^ for polymer containing sample (**MNP@PAAA-FA**). This on the surface without pore filling or blockage. The porosity analysis reveals that the **MNP** is mesoporous with a medium pore size of 15 nm and tight pores’ size distribution (Figure S2). For **MNP@PAAA-FA** instead, alongside the 14 nm pores, Figure S2 reveals the existence of larger pores (25 nm and 50 nm) which are not part of the intrinsic material’s porosity. These pores can be formed between magnetite nanoparticles aggregated by the polymer in a nano-agglomerated material.

#### 3.2.2. Effect of Initial CV Concentrations and Contact Time

**Figure 5:**
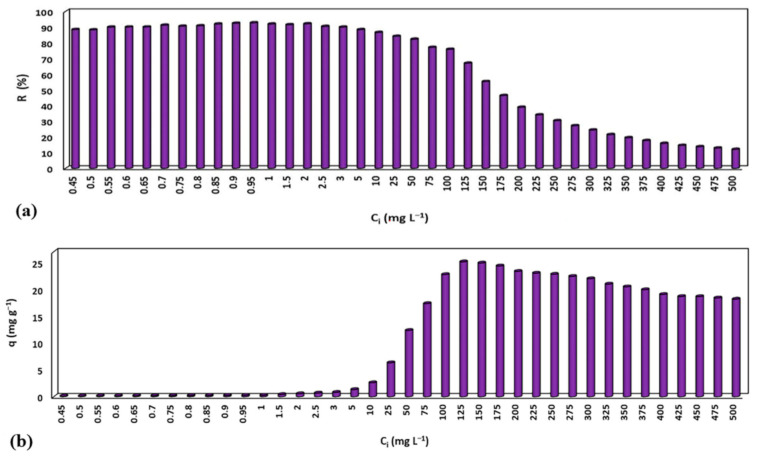
Effect of initial CV concentrations (0.45–500 mg L^−1^) on the removal efficiency (**a**) and adsorption capacity (**b**) of **MNP@PAAA-FA** determined by UV-VIS spectroscopy at a spectral resolution of 1 nm and 591 nm wavelength.

The influence of initial CV concentrations on the adsorption capacity/removal efficiency of **MNP@PAAA-FA** was investigated (Figure 5a). The obtained removal efficiencies were very high (82.29%–92.44%) for initial dye concentrations from 0.45–500 mg L^−1^, followed by a linear decrease to 38.91% at 200 mg L^−1^ and approaching the saturation limit at 500 mg L^−1^ (12.11%). This fact highlights an inverse proportionality between the CV concentration in the aqueous solution and the removal efficiency, explained by an inhibition that most likely arises due to the higher number of CV molecules gathering on the surface of **MNP@PAAA-FA** and competing for the same available active sites. Nonetheless, the adsorption capacity is directly proportional to the initial CV concentration, achieving a maximum value of 25.12 mg g^−1^ at 125 mg L^−1^ (Figure 5b). However, a small diminution from 24.89 mg g^−1^ to 18.17 mg g^−1^ can be noticed between 150 mg L^−1^ and 500 mg L^−1^.

**Figure 6:**
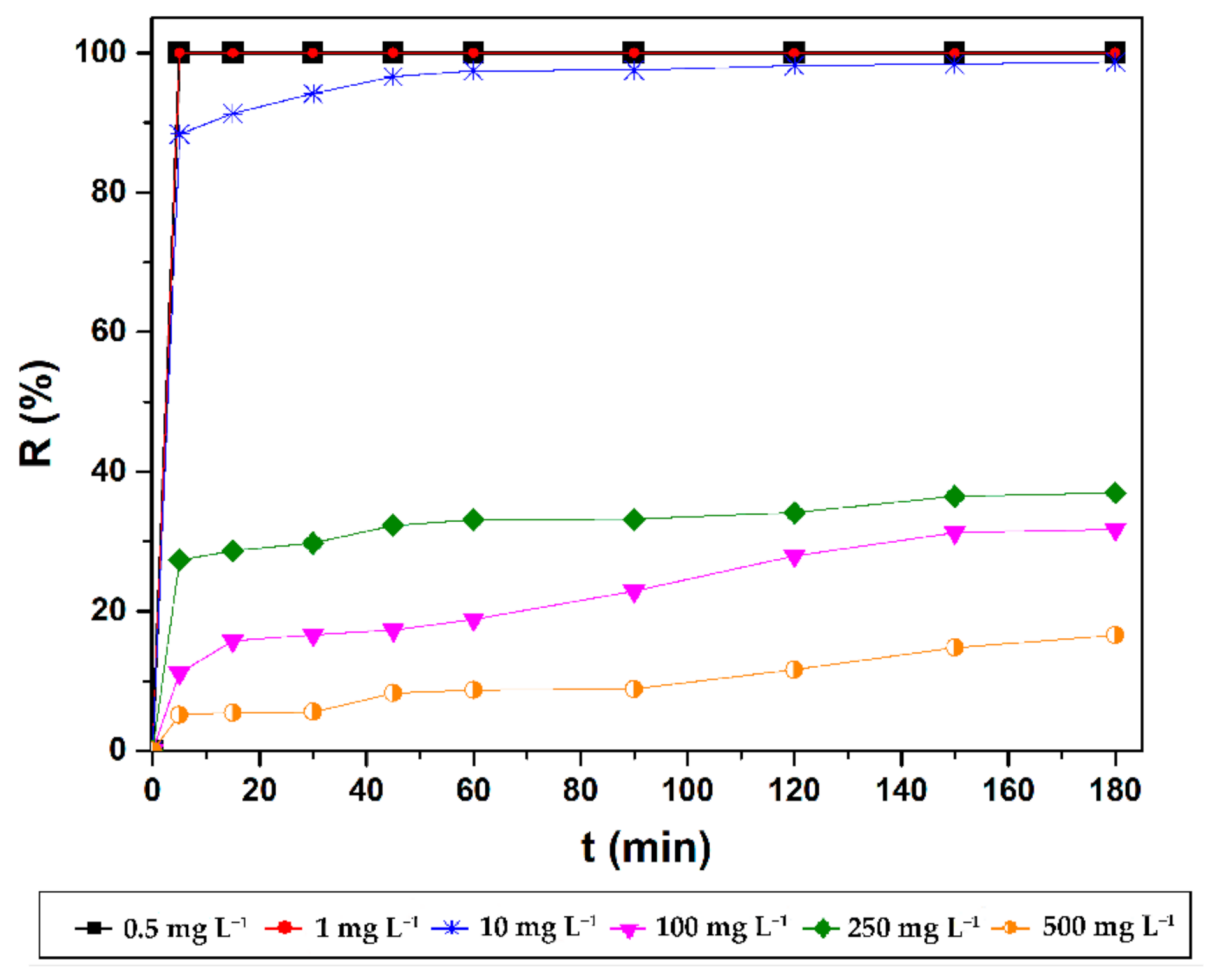
Effect of contact time on CV removal efficiency of **MNP@PAAA-FA.**

**Figure 7:**
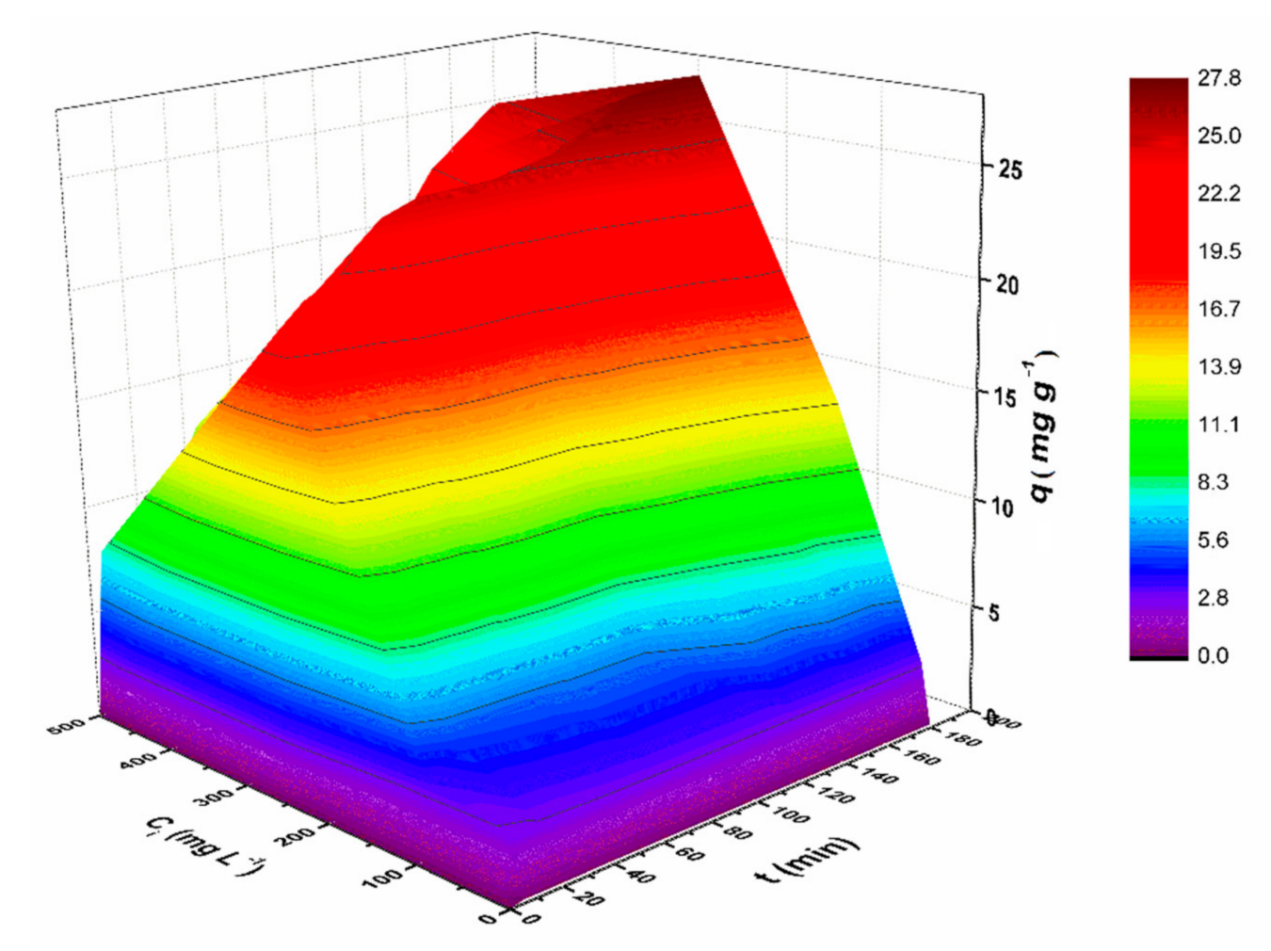
The 3D Adsorption model of combined effects of independent variables on the adsorption capacity of CV by **MNP@PAAA-FA**

Furthermore, the influence of contact time on the adsorption process evolution was also investigated. As it can be seen in Figure 6, the adsorption of CV dye onto the magnetic nanostructures was very fast at the beginning (first 5 min), the removal efficiency reaching 100% for low initial concentrations (0.5 mg L^−1^ and 1 mg L^−1^) due to the abundant availability of active binding sites. CV retention followed a gradual tendency at higher concentrations due to the depletion of these active sites in time. The experimental data indicated a maximum adsorption capacity within 180 min (Figure S3). Thus, it can be stated that the presence of various functional groups, the structure and the surface area of the magnetic nanostructures influenced the CV retention. Figure 7 summarizes the effects of the two investigated independent variables (contact time and initial CV concentration) on the sorption capacity of **MNP@PAAA-FA**. As expected, adsorption capacity increased proportionally with both studied parameters.

Table 1 presents a short comparison between the CV adsorption capacities reported in the scientific literature for various magnetic materials and those obtained for **MNP@PAAA-FA** in the current study. The magnetic nanomaterial presented in the current study shows good adsorption capacity compared to other magnetic adsorbents.

#### 3.2.3. Equilibrium Studies on CV Dye Adsorption by **MNP@PAAA-FA**

Five adsorption isotherms were used to examine the interactions between CV molecules and **MNP@PAAA-FA** surface: Langmuir [33], Freundlich [30], Temkin [34], Dubinin-Radushkevich [31] and Khan [32]. The linear forms of the considered CV adsorption isotherms are presented in Figure S4, while the equilibrium coefficients and descriptive statistics are shown in Tables S3 and S4, respectively. Anscombe’s quartet was also considered in the current study (Figure S5).

The adsorption equilibrium study indicates that the sorption behaviour of CV on **MNP@PAAA-FA** satisfies Langmuir, Freundlich and Dubinin-Radushkevich assumptions. The data fitted very well on the first linear plot of the Langmuir isotherm model (adj. R^2^ = 0.99), highlighting a 19.45 mg g^−1^ maximum sorption capacity of CV on the newly synthesized material (Table S3). Moreover, the R_L_ values obtained were in the 0.001–0.56 range, which is less than 1, showing that the adsorption of CV onto the **MNP@PAAA-FA** surface is a favourable process, normally occurring under the given conditions. It can also be noticed that for initial CV concentrations between 400–500 mg L^−1^, the R_L_ values (0.001) were very close to 0, indicating that the adsorption process is getting too strong (becoming irreversible) (Figure S6).

In the case of Freundlich isotherm (adj. R^2^ = 0.94), the value obtained for n (1.76) is higher than 1, thus the 1/n ratio is less than 1 (0.57), suggesting that the CV adsorption is a favourable process, with an intense bond between adsorbent and adsorbate. The heat of adsorption (K_T_) determined from the Temkin isotherm is positive, indicating an exothermic character of the CV adsorption process. According to the Dubinin-Radushkevich isotherm, the average free energy value (2.36 kJ mol^−1^) is less than 8 kJ mol^−1^, demonstrating that the CV adsorption onto the **MNP@PAAA-FA** has been carried out physically.

**Figure 8:**
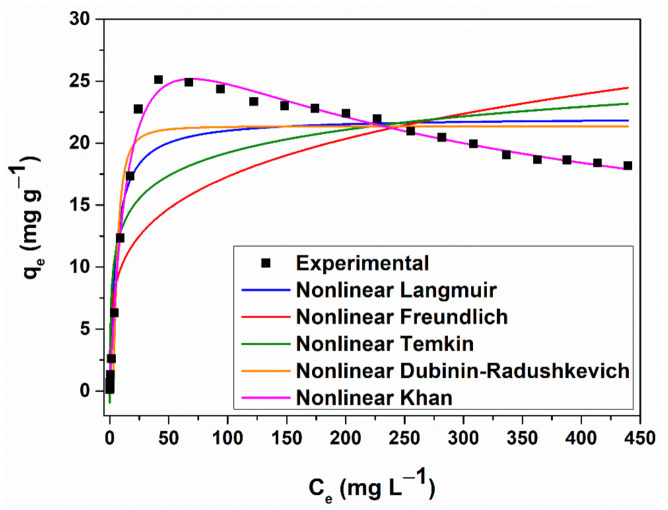
Nonlinear forms of the equilibrium isotherms obtained for CV sorption onto **MNP@PAAA-FA** Considering both the lowest standard deviation value and the highest coefficient of determination, it can be stated that Khan isotherm model fits the best CV adsorption process. Thus, the αK value (1.38) is around 1, indicates that the CV adsorption process approaches the Langmuir isotherm at lower concentrations, while at higher values, this model reduces to the Freundlich isotherm.

The nonlinear analysis was used to avoid estimation errors resulting from the linear regression method (Figure 8). Comparing the adjusted correlation coefficients obtained for each of the considered nonlinear isotherm models (Table S5), the data can be fitted following the next sequence: Khan (best fit) (adj. R^2^ = 0.99), Dubinin-Radushkevich (adj. R^2^ = 0.97), Langmuir (adj. R^2^ = 0.96), Temkin (adj. R^2^ = 0.89), Freundlich (adj. R^2^ = 0.80). On the other hand, comparing the standard deviation values, the fitting degree follows a similar sequence: Khan (S_D_ = 0.46), Dubinin-Radushkevich (S_D_ = 1.85), Langmuir (S_D_ = 2.16), Temkin (S_D_ = 3.39), Freundlich (S_D_ = 4.69).

#### 3.2.4. Kinetic Studies on CV Dye Adsorption by **MNP@PAAA-FA**

**Figure 9:**
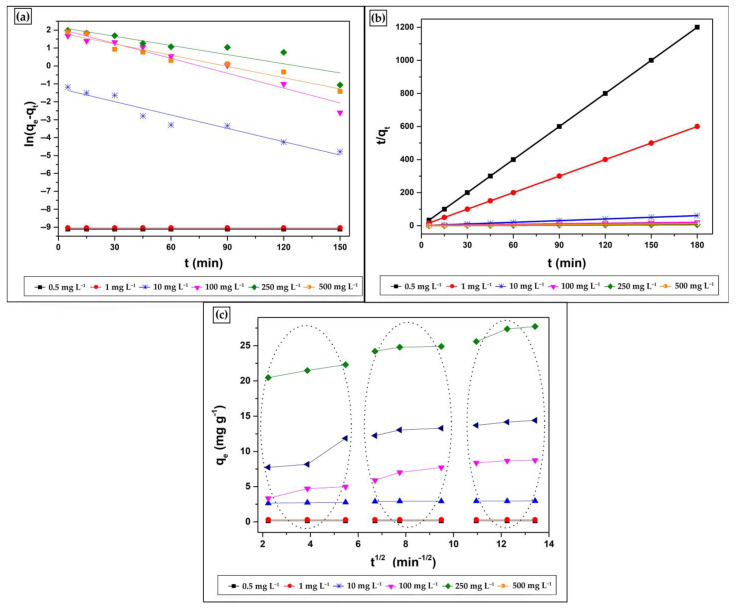
Pseudo-First Order (**a**), Pseudo-Second Order (**b**) and Weber-Morris Intra-Particle Diffusion (**c**) kinetic models used to describe CV adsorption on **MNP@PAAA-FA.** The initial CV concentrations were 0.5, 1, 10, 100, 250 and 500 mg L^−1^ using 100 mg of adsorbent at 298 K and 700 rpm.

Three kinetic models [37,38,39] were considered (Figure 9) to optimise the adsorption process. Thus, to determine the kinetic model that best portrays the CV adsorption on **MNP@PAAA-FA**, a linear regression was performed. Table 2 summarizes the adsorption rate constants of pseudo-first-order (k_1_), pseudo-second-order (k_2_) kinetic models, the Weber-Morris diffusion coefficient (k_ipd_), the calculated (q_e_) and experimental amounts (q_e1_, q_e2_) of CV adsorbed, and the correlation coefficients (R^2^) obtained. Considering both the low values of R^2^ and the large difference between the experimental and the estimated values, it can be stated that the sorption mechanism cannot be categorized as pseudo-first-order. Instead, the pseudo-second-order kinetic model was more suitable for describing the CV adsorption process on **MNP@PAAA-FA** (adj. R^2^ = 0.99–1), suggesting the occurrence of a second-order mechanism, where chemisorption represents the rate-limiting step. This points towards a balance between the adsorption sites’ filling rate and the square number of unoccupied sites. Furthermore, plotting q_t_ versus t^1/2^ does not have a linear trend for CV concentrations higher than 10 mg L^−1^ over the whole-time range considered in the current study. Since the line from the initial step does not intersect the origin, it demonstrates that the intra-particle diffusion process is not the sole mechanism engaging in the adsorption and that other processes can also contribute to the rate-determining step. The adsorption assays for 0.50 mg L^−1^, 1 mg L^−1^ and 10 mg L^−1^ CV concentrations highlight an instantaneous adsorption stage (external surface adsorption). However, at higher concentrations, competitive adsorption occurs in the solution: the first portion (between 2–5.50 min^−1/2^) represents the instantaneous adsorption stage (film diffusion—the CV molecules diffuse throughout the solution to the **MNP@PAAA-FA** surface), the second segment (between 6–9.50 min^−1/2^) follows a progressive adsorption pattern (with intra-particle diffusion as the rate-controlled step) and the third section (between 11–13.50 min^−1/2^) depicts the final equilibrium stage (when intra-particle diffusion decreases due to the lower number of free adsorption sites and the CV concentration diminution). Thus, although three processes control the sorption rate at higher CV concentrations, only one is preponderant at any particular time phase. The intra-particle diffusion constant (I) values suggest that surface diffusion presents a minor contribution as the rate-limiting stage in the entire sorption process.

#### 3.2.5. **MNP@PAAA-FA** Regeneration Study

**Figure 10:**
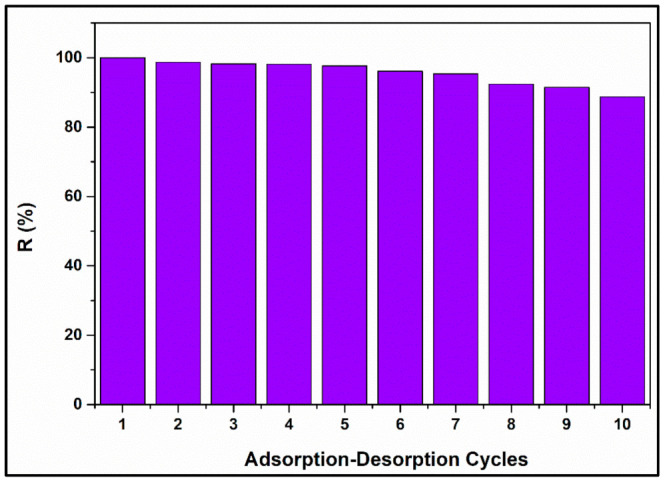
Reusability of **MNP@PAAA-FA** after conducting 10 cycles of adsorption-desorption.

A study on the reusability of **MNP@PAAA-FA** was also conducted. Figure 10 shows excellent CV adsorption efficiency onto **MNP@PAAA-FA** after performing 10 cycles of adsorption-desorption. Removal efficiency gradually decreased from 100% to 97.63% in the first five cycles, reaching a minimum of 88.74% after the 10th regeneration stage.

## 4. Conclusions

A novel form of magnetic nanostructures prepared from poly(benzofuran-*co*-arylacetic acid) was developed through “green chemical methods”, characterized and further applied in crystal violet adsorption experiments. Anchoring **PAAA-FA** on the **MNP** surface was achieved by avoiding any organic solvents or additional catalyst. The resulting material includes various functional groups which provide many opportunities to link or immobilize contaminants. Experimental adsorption records were investigated employing five isotherm models (Langmuir, Freundlich, Temkin, Dubinin-Radushkevich and Khan) in both linear and nonlinear regression and three kinetic models (Pseudo-first order, Pseudo-second order kinetics and Weber-Morris Intra-particle Diffusion). The Khan nonlinear regression investigation and the Pseudo-second order kinetics had the best performances. Maximum removal efficiencies up to 92.75% were obtained after only 30 min. Considering both the equilibrium and the kinetic studies, it can be stated that CV adsorption occurs very fast in the first 40 min, while after 180 min the equilibrium is reached. Based on the obtained removal efficiencies, we can conclude that due to the complex structure of **MNP@PAAA-FA** it can interconnect and retain the CV molecules dye through several types of interactions as electrostatic, π–π stacking interaction, etc., playing an essential role in the adsorption mechanism. The batch experiments regarding CV adsorption on **MNP@PAAA-FA** proved good removal efficiencies and sorption capacities, thus recommending them as good candidates for further applications in wastewater decontamination.

## Figures and Tables

**Table 1:** Summary of CV adsorption capacities reported in the literature for different adsorbent materials.

Adsorbent Material	q (mg g^−1^)	Reference
Magnetic NaY Zeolite	2.00	[42]
Magnetic Calcium Ferrite Nanoparticles	0.80	[21]
Nanomagnetic Iron Oxide	16.50	[20]
Magnetic Charcoal	10.00	[43]
Magnetic Carbon-Iron Oxide Nanocomposite	81.70	[44]
Bentonite Iron Cobalt Nanocomposite	13.00	[45]
Magnetite Nanoparticles Loaded Azolla	25.00	[46]
Carboxymethyl Chitosan-Based Magnetic Nanocomposite	72.40	[47]
**MNP@PAAA-FA**	25.10	Current Study

**Table 2:** Results of the kinetic models for CV adsorption on **MNP@PAAA-FA** (Ci = 0.5–500 mg L^−1^, 100 mg material, 298 K, 700 rpm).

C_i_ CV (mg L^−1^)	q_e_ (mg g^−1^)	Pseudo-First Order			Pseudo-Second Order			Morris Weber Intra-Particle Diffusion		
		k_1_ (min^−1^)	q_e1_ (mg g^−1^)	adj. R^2^	k_2_ (g mg^−1^ min^−1^)	q_e2_ (mg g^−1^)	adj. R^2^	k_ipd_ (mg g^−1^ min^−1/2^)	I	adj. R^2^
**0.5**	1.50 × 10^−1^	1.60 × 10^−17^	11.00 × 10^−5^	1	5.90 × 10^−13^	1.50 × 10^−1^	1	0	1.50 × 10^−1^	1
**1**	3.00 × 10^−1^	3.20 × 10^−17^	12.00 × 10^−5^	1	5.90 × 10^−13^	3.00 × 10^−1^	1	0	3.00 × 10^−1^	1
**10**	29.60 × 10^−1^	24.80 × 10^−3^	28.70 × 10^−1^	0.93	7.40 × 10^−1^	29.80 × 10^−1^	0.99	2.70 × 10^−2^	26.40 × 10^−1^	0.81
**100**	87.40 × 10^−1^	27.60 × 10^−3^	80.30 × 10^−1^	0.94	49.00 × 10^−1^	96.80 × 10^−1^	0.99	50.30 × 10^−2^	25.70 × 10^−1^	0.96
**250**	2.80 × 10^−1^	17.00 × 10^−3^	87.30 × 10^−1^	0.81	1.50 × 10^−1^	2.80 × 10^−1^	0.99	64.20 × 10^−2^	1.90 × 10^−1^	0.96
**500**	1.40 × 10^−1^	20.90 × 10^−3^	64 × 10^−1^	0.94	1.10 × 10^−1^	1.50 × 10^−1^	0.99	59.40 × 10^−2^	73.00 × 10^−1^	0.82

## Data Availability

No new data were created or analyzed in this study. Data sharing does not apply to this article.

## Supplementary Materials

The following are available online at https://www.mdpi.com/2079-4991/11/3/679/s1, Table S1: Equilibrium Isotherm Models, Table S2: Kinetic Models, Table S3: Results of the adsorption isotherm models based on the linear regression analysis for CV on **MNP@PAAA-FA** (Ci = 0.45–500 mg L^−1^, 10 mg material, 298 K, 700 rpm, 30 min), Table S4: Summary of descriptive statistics applied on adsorption experimental data, Table S5: Results of the adsorption isotherm models based on the nonlinear regression analysis for CV on **MNP@PAAA-FA** (Ci = 0.45–500 mg L^−1^, 10 mg material, 298 K, 700 rpm, 30 min), Figure S1: X-ray powder diffraction (XRPD) patterns of magnetic nanostructure **MNP@PAAA-FA,** Figure S2: N_2_ adsorption–desorption isotherms and pore size distribution of **MNP** (a,c) and **MNP@PAAA-FA** (b,d), Figure S3: Effect of contact time on CV adsorption capacity of **MNP@PAAA-FA**, Figure S4: Linear forms of the Langmuir (a), Freundlich (b), Temkin (c) and Dubinin-Radushkevich (d) adsorption equilibrium isotherms, Figure S5: Anscombe’s quartet applied to our adsorption experimental data, Figure S6: Separation factor RL versus initial concentration for the adsorption of CV onto **MNP@PAAA-FA**.
