# Supplementary material for: Effective Removal of Crystal Violet Dye Using Neoteric Magnetic Nanostructures Based on Functionalized Poly(Benzofuran-co-Arylacetic Acid): Investigation of the Adsorption Behaviour and Reusability
Source: Nanomaterials (Basel). 2021 Mar 9;11(3):679. doi: 10.3390/nano11030679 (PMC7999123; doi:10.3390/nano11030679)
Supplement: Supplementary file 1 [file nanomaterials-11-00679-s001.pdf]

nanomaterials

Article

Effective removal of Crystal Violet dye using neoteric magnetic nanostructure based on functionalized poly(benzofuran-*co*-arylacetic acid): Investigation of the adsorption behaviour and reusability

Iolanda-Veronica Ganea [1,2], Alexandrina Nan [1,*], Călin Baciu [2], Rodica Turcu [1,*]

Supplementary Materials

Section A. Methodology regarding the adsorption equilibrium and kinetics experiments

The Langmuir isotherm model [1] pretends the adsorption leads to a monolayer surface, without interactions among the adsorbate molecules, while the Freundlich [2] and Dubinin-Radushkevich [3] models are suitable for both monolayer and multilayer adsorption processes, considering that the sorption develops on a heterogeneous surface. Temkin isotherm [4] states that the molecules adsorption energy proportionally declines with the surface coverage because of adsorbate–adsorbent interchanges. Khan model [5] represents another theoretical equation developed from Langmuir isotherm (Table S1).

Table S1. Equilibrium Isotherm Models

Isotherm Models	Nonlinear Forms	Linear Forms	Plot	Reference
**Langmuir**	$q_e = \frac{q_{max} \times K_L \times C_e}{1 + K_L \times C_e}$	$\frac{C_e}{q_e} = \frac{C_e}{q_{max}} + \frac{1}{K_L \times q_{max}}$	C_e/q_e versus C_e	[1]
**Freundlich**	$q_e = K_F \times C_e^{1/n}$	$\ln q_e = \ln K_F + \frac{1}{n} \times \log C_e$	$\ln(q_e)$ versus $\ln(C_e)$	[2]
**Dubinin-Radushkevich**	$q_e = q_{max} \times \exp(-K_{DR} \times \varepsilon^2)$	$\ln q_e = \ln q_{max} - K_{DR} \times \varepsilon^2$ where $\varepsilon = R \times T \times \ln\left(1+\frac{1}{C_e}\right)$ $E = \frac{1}{\sqrt{2 \times K_{DR}}}$	$\ln(q_e)$ versus ε^2	[3]
**Temkin**	$q_e = B \times \ln(A_T \times C_e)$ where $B = \frac{R \times T}{K_T}$	$q_e = B \times \ln A_T + B \times \ln C_e$ where $B = \frac{R \times T}{K_T}$	q_e versus $\ln(C_e)$	[4]
**Khan**	$q_e = \frac{q_S \times b_K \times C_e}{(1 + b_K \times C_e)^{\alpha_K}}$	–	–	[5]

where: q_e (mg g^{-1}) is the experimental amount of pollutant adsorbed per unit mass of material; C_e (mg L^{-1}) is the contaminant concentration at equilibrium; q_{max} (mg g^{-1} per unit mass) represents the maximum amount of adsorbate; K_F is the Freundlich constant (showing the adsorption capacity); n represents the adsorption intensity (n < 1 proves a chemical adsorption

process, while n > 1 denotes a physical adsorption process); q_s is the theoretical isotherm saturation capacity (mg g^{-1}); ε and K_{DR} (mol^2 kJ^{-2}) are the Dubinin–Radushkevich isotherm constants; R is the universal gas constant (8.314 J mol^{-1} K^{-1}); T is the temperature (K); E (kJ mol^{-1}) is the mean free energy (if E is between 8–16 kJ mol^{-1}, the adsorption process is of chemical type and if E < 8 kJ mol^{-1}, the adsorption proceeds physically) ; B (J mol^{-1}) is a constant associated with the adsorption heat (indicating the adsorption potential of the material); K_T and A_T (L g^{-1}) are the Temkin isotherm constant and the equilibrium binding constant, respectively; α_K and b_K are the exponent and constant of the Khan isotherm model.

The pseudo-first order model was developed by Lagergren in 1898 [6] and is the most common adsorption kinetic model. Ho and Mckay [7] proposed the pseudo-second order model and takes into consideration the sorption capacity of the solid phase, allowing the calculation of the performance over the entire range of adsorption. The intra-particle diffusion model was first introduced by Weber and Morris [8] and takes into consideration the pore size and the surface diffusion (Table S2).

Table S2. Kinetic Models

Kinetic Model	Nonlinear Form	Linear Form	Reference
**Pseudo-first-order**	$\frac{dq_t}{dt} = k_1 \times (q_{e1} - q_t)$	$\log\left(q_{e_1} - q_{t_1}\right) = \log q_{e_1} - \left(\frac{k_1}{2.303}\right) \times t$	[6]
**Pseudo-second-order**	$\frac{dq_t}{dt} = k_2 \times (q_{e2} - q_t)^2$	$\frac{t}{q_{t_2}} = \frac{1}{k_2\, q_{e_2}^{\,2}} + \frac{t}{q_{e_2}}$	[7]
**Morris Weber intra-particle diffusion**	–	$q_t = k_{ipd} \times t^{1/2} + I$	[8]

where: q_{e_1}, q_{e_2} (mg g^{-1}) represent the experimental amount of pollutant adsorbed at the equilibrium state; q_t, q_{t_1}, q_{t_2} (mg g^{-1}) are amount of pollutant adsorbed at time t (min); k_1 (min^{-1}) and k_2 (g mg^{-1} min^{-1}) represent the adsorption rate constants of the pseudo-first-order and pseudo-second-order adsorption models; k_{ipd} (mg g^{-1} min^{-1}) is a measure of the diffusion coefficient and I is the intra-particle diffusion constant (that describes the thickness of the boundary layer). K_1, K_2 parameters and q_e were calculated from the intercepts and slopes obtained by plotting $\ln(q_e - q_t)$ versus t and t/q_t versus t, respectively, while K_{ipd} and I were determined from the plot of q_t versus $t^{1/2}$.

Section B. Synthesis and characterization of magnetic nanostructure based on **MNP@PAAA-FA**

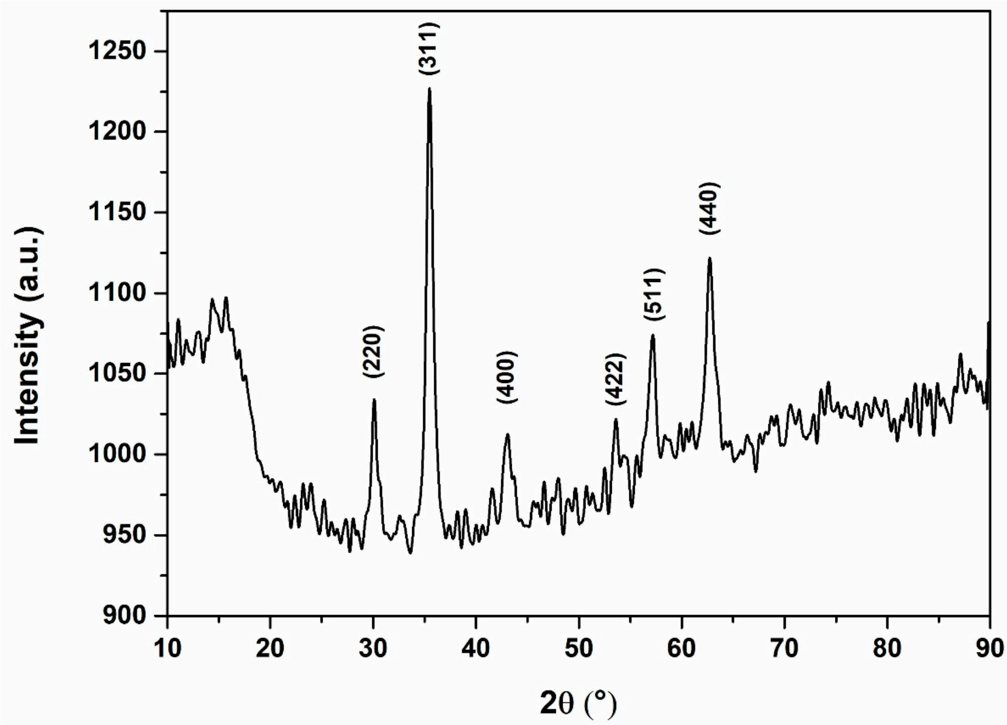

Figure S1. *X*-ray powder diffraction (XRPD) patterns of magnetic nanostructure **MNP@PAAA-FA**

Section C. Adsorption tests on the new magnetic nanostructure based on poly(benzofuran-co-arylacetic acid) functionalized with folic acid

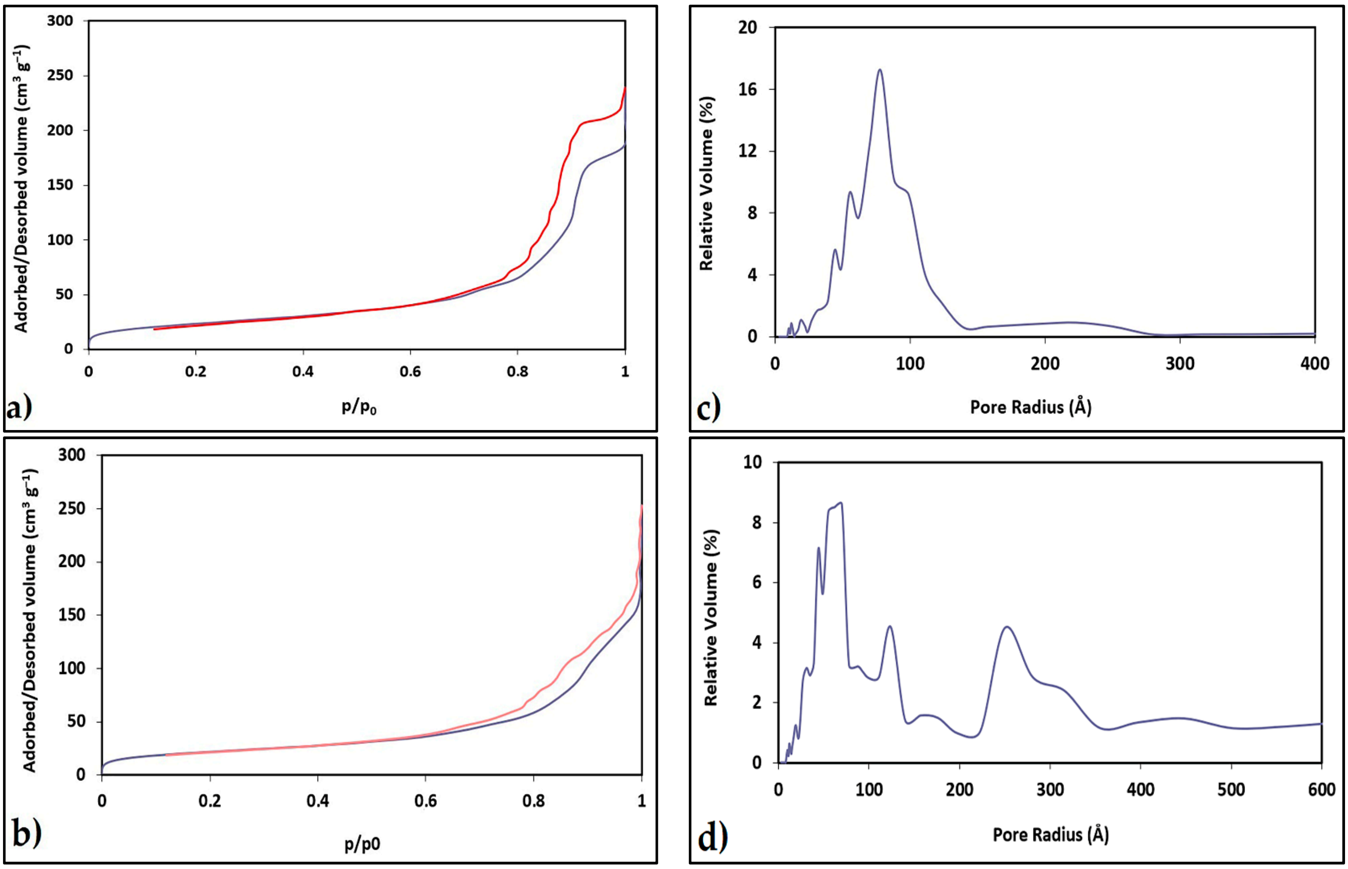

Figure S2. N_2 adsorption–desorption isotherms and pore size distribution of **MNP (a,c)** and **MNP@PAAA-FA (b,d).**

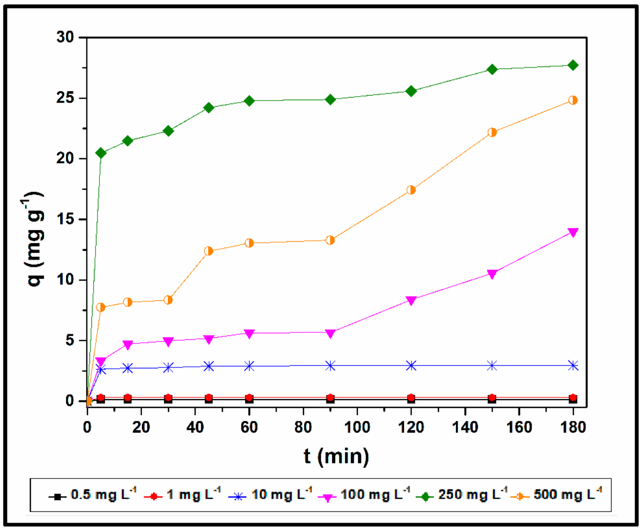

Figure S3. Effect of contact time on CV adsorption capacity of **MNP@PAAA-FA**

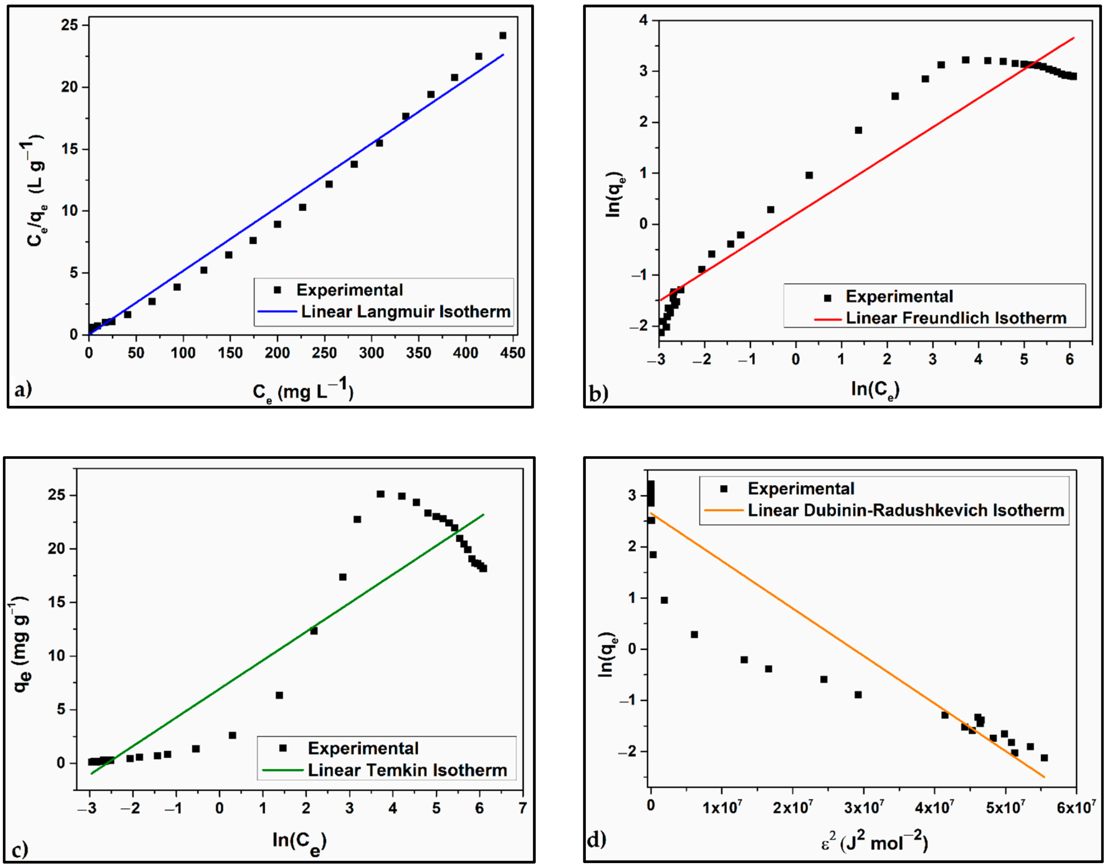

Figure S4. Linear forms of the Langmuir **(a)**, Freundlich **(b)**, Temkin **(c)** and Dubinin-Radushkevich **(d)** adsorption equilibrium isotherms

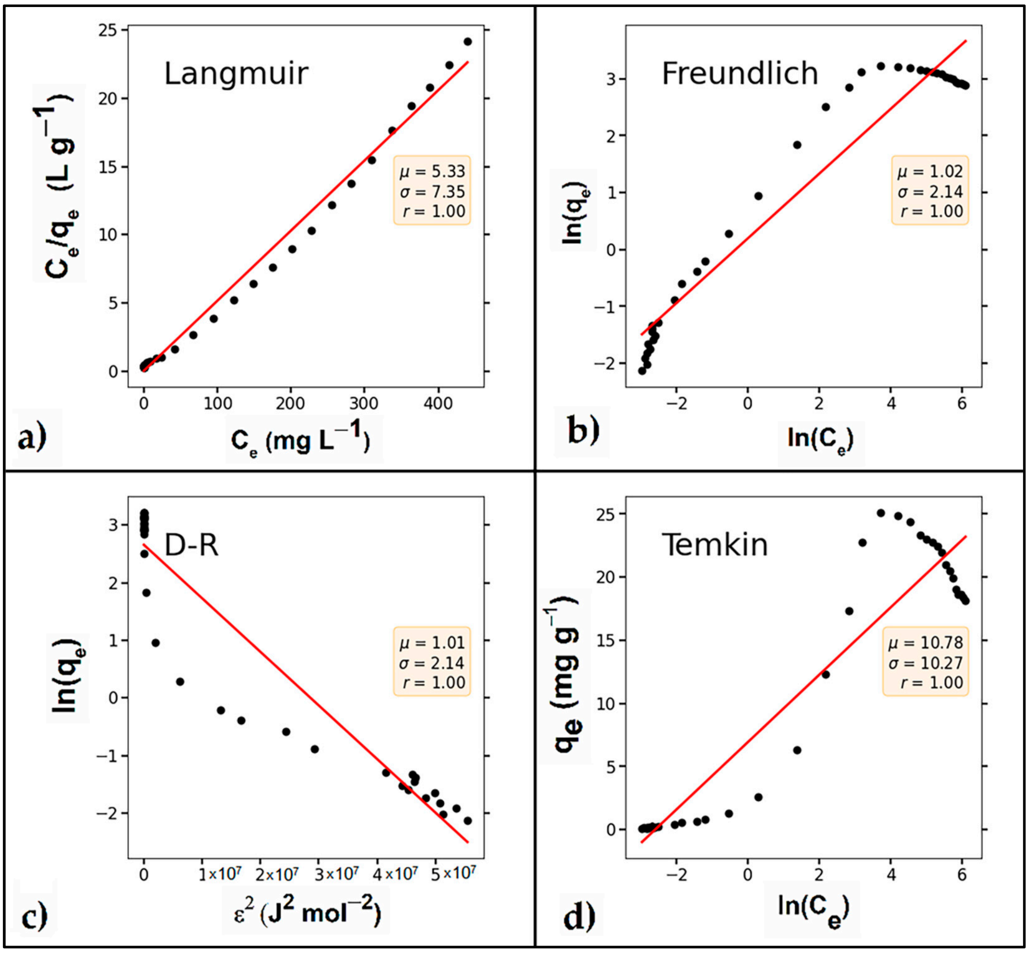

Figure S5. Anscombe's quartet applied to our adsorption experimental data

Table S3. Results of the adsorption isotherm models based on the linear regression analysis for CV on **MNP@PAAA-FA** (C_i = 0.45–500 mg L^{-1}, 10 mg material, 298 K, 700 rpm, 30 min)

Isotherm Model	Equilibrium Coefficients	
Langmuir	K_L (L mg^{-1})	1.7
	q_{max} (mg g^{-1})	19.5
	R^2	0.99
Freundlich	K_F	1.2
	n	1.8
	R^2	0.94
Temkin	K_T	928.4
	A_T (L g^{-1})	13.4
	R^2	0.89
Dubinin-Radushkevich	q_{max} (mg g^{-1})	14.3
	K_{DR} (mol^2 kJ^{-2})	$9.3{\cdot}10^{-12}$
	E (kJ mol^{-1})	2.4
	R^2	0.90

Table S4. Summary of descriptive statistics applied on adsorption experimental data.

Statistical Parameter	Equilibrium Isotherm			
	Langmuir	Freundlich	Dubinin-Radushkevich	Temkin
Mean	5.33	1.02	1.01	10.78
Variance	55.54	4.71	4.71	108.39
Coefficient of Variation	1.40	2.14	2.14	0.97
Median	0.67	2.18	2.18	9.33
Residual Sum of Squares	19.12	10.64	16.54	414.29

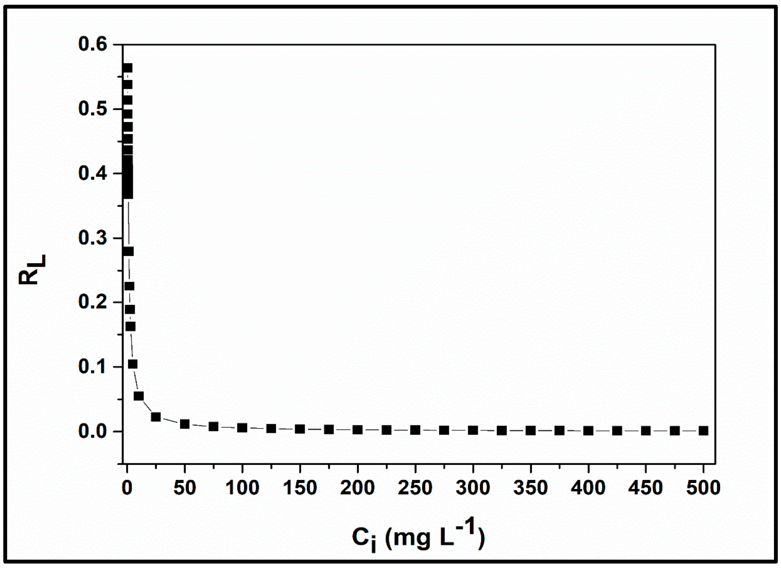

Figure S6. Separation factor R_L versus initial concentration for the adsorption of CV onto **MNP@PAAA-FA**

Table S5. Results of the adsorption isotherm models based on the nonlinear regression analysis for CV on **MNP@PAAA-FA** (Ci = 0.45–500 mg L^{-1}, 10 mg material, 298 K, 700 rpm, 30 min)

Isotherm Model	Equilibrium Coefficients	
Langmuir	K_L (L mg^{-1})	0.2
	q_{max} (mg g^{-1})	22.1
	R^2	0.96
	S_D	2.2
Freundlich	K_F	5.9
	n	4.3
	R^2	0.80
	S_D	4.7
Temkin	K_T	928.4
	A_T (L g^{-1})	13.4
	R^2	0.89
	S_D	3.4
Dubinin-Radushkevich	q_{max} (mg g^{-1})	21.4
	K_{DR} (mol^2 kJ^{-2})	$5 \cdot 10^{-6}$
	R^2	0.97
	S_D	1.9
Khan	q_{max} (mg g^{-1})	57.1
	α_K	1.4
	b_K	0.04
	R^2	0.99
	S_D	0.5

References

1. Langmuir, I. The constitution and fundamental properties of solids and liquids. *J. Am. Chem. Soc.* **1918**, *40*, 1361–1403.
2. Freundlich, H. Über die absorption in lösungen. *Zeitschrift für Physikalische Chemie- Stöchiometrie und verwandschaftslehre* **1907**, *57*, 385–470.
3. Dubinin, M.M.; Radushkevich, L.V. The equation of the characteristic curve of activated charcoal. *Proc. Acad. Sci. USSR Phys. Chem. Sect.* **1947**, *55*, 331–337.
4. Temkin, M.J.; Pyzhev, V. Kinetics of ammonia synthesis on promoted iron catalysts. *Acta Physicochim. URSS*. **1940**, *12*, 217–222.
5. Khan, A.R.; Ataullah, R.; Al-Haddad, A. Equilibrium adsorption studies of some aromatic pollutants from dilute aqueous solutions on activated carbon at different temperatures. *J. Colloid Interface Sci.* **1997**, *194*, 154–165.
6. Lagergren, S.; Sven, K. Zur theorie der sogennanten adsorptiongeloster stoffe. *Kungliga Sevenska Vetenskapsakademiens. Handlingar*. **1898**, *24*, 1–39.
7. Ho, Y.S.; McKay, G. The kinetics of sorption of divalent metal ions onto sphagnum moss peat. *Water Res*. **2000**, *34*, 735–742.
8. Weber, W.J.; Morris, J.C. Kinetic of adsorption on carbon from solution. *Am. Soc. Civ. Eng*. **1963**, *89*, 1–40.
